# Cognitive benefits of using non-invasive compared to implantable neural feedback

**DOI:** 10.1038/s41598-022-21057-y

**Published:** 2022-10-06

**Authors:** Lauren Chee, Giacomo Valle, Greta Preatoni, Chiara Basla, Michele Marazzi, Stanisa Raspopovic

**Affiliations:** Laboratory for Neuroengineering, Department of Health Science and Technology, Institute for Robotics and Intelligent Systems, ETH Zürich, 8092 Zürich, Switzerland

**Keywords:** Somatic system, Biomedical engineering

## Abstract

A non-optimal prosthesis integration into an amputee’s body schema suggests some important functional and health consequences after lower limb amputation. These include low perception of a prosthesis as a part of the body, experiencing it as heavier than the natural limb, and cognitively exhausting use for users. Invasive approaches, exploiting the surgical implantation of electrodes in residual nerves, improved prosthesis integration by restoring natural and somatotopic sensory feedback in transfemoral amputees. A non-invasive alternative that avoids surgery would reduce costs and shorten certification time, significantly increasing the adoption of such systems. To explore this possibility, we compared results from a non-invasive, electro-cutaneous stimulation system to outcomes observed with the use of implants in above the knee amputees. This non-invasive solution was tested in transfemoral amputees through evaluation of their ability to perceive and recognize touch intensity and locations, or movements of a prosthesis, and its cognitive integration (through dual task performance and perceived prosthesis weight). While this managed to evoke the perception of different locations on the artificial foot, and closures of the leg, it was less performant than invasive solutions. Non-invasive stimulation induced similar improvements in dual motor and cognitive tasks compared to neural feedback. On the other hand, results demonstrate that remapped, evoked sensations are less informative and intuitive than the neural evoked somatotopic sensations. The device therefore fails to improve prosthesis embodiment together with its associated weight perception. This preliminary evaluation meaningfully highlights the drawbacks of non-invasive systems, but also demonstrates benefits when performing multiple tasks at once. Importantly, the improved dual task performance is consistent with invasive devices, taking steps towards the expedited development of a certified device for widespread use.

## Introduction

Sensory loss due to amputation or nerve damage breaks the sensorimotor control loop and has both negative functional and health effects. In lower-limb amputees (LLA), these can include asymmetric gait and risk of falling as a result of reduced balance^1^ leading to osteoarthritis in the intact limb and osteoporosis in the residual limb^2^ as well as higher metabolic consumption when walking^3^ with consequent higher cardiovascular morbidity^4^. LLAs also perceive the prosthesis as excessively heavy (despite it being half the weight of a healthy leg)^5^ which can be linked to cognitive deficits such as lower prosthesis embodiment^6^, cognitive integration^5^ and overall multisensory integration^7^. In addition to these physical effects, reduced confidence affects mobility and in turn the ability to participate in social activities^8^, reducing overall quality of life.

Restoration of lost sensations through neuro-integrated leg prostheses in LLAs^9,10^, specifically transfemoral (above the knee) amputees, has demonstrated important functional and cognitive benefits^5,11,12^. In these studies, lost sensory information was restored through the implantation of transversal intrafascicular multichannel electrodes (TIMEs^13^) in the residual nerves in the thigh. This allowed for real time encoding of force and knee angle information from the prosthetic leg and direct stimulation of the residual nerves evoking close to natural tactile sensations in the phantom foot and proprioceptive-like contraction of the phantom calf^12^. These invasive (i.e. requiring a surgery) solutions returned useful foot-ground and prosthetic flexion information to subjects which resulted in improved mobility, agility, prosthesis’ embodiment, and dual task performance, while reducing falls. This information also reduced metabolic consumption and phantom-limb pain^12,13^ all of which contribute to a significant improvement in the health and quality of life of a LLA, and are thus incredibly impactful. These benefits are most likely connected to the restored CNS schema of the lost limb due to the physiologically plausible peripheral sensory drive^12,14^.

Similar invasive approaches (implanted electrodes^15^ or surgery to connect paired agonist and antagonist muscles^16^) in transtibial (below the knee) amputees have also shown promising results, being able to elicit somatotopic (i.e. referred on the phantom limb) tactile sensations in the phantom foot/leg^10^ as well as proprioceptive sensations^16^. More recently, another invasive method (i.e. mechano-neuro-interface) that involves implanting an electrode over a muscle actuator on the afferent nerve^17^ has shown promise as an amputation technique that demonstrates improved motor control and position differentiation when compared with traditional amputation methods in transtibial amputees^18^.

This wide set of meaningful benefits in both transtibial and transfemoral amputees is undercut by the requirement of a surgical procedure in order to implant electrodes^11,15^ in the nerves or connect the agonist/antagonist muscles^16–18^. These surgical procedures may lead to additional surgeries after amputation that patients would prefer not to undergo unless strictly necessary^19^. The operation is also an expensive and time-consuming process since the surgeon must be specifically trained to perform it. Avoiding a surgery and its associated complications and costs is appealing not only for the patient, but also for the healthcare system. Non-invasive sensory feedback systems that do not require a surgery have shown some interesting preliminary results^20–23^ but have not yet been able to demonstrate consistent and well supported functional benefits that would improve patients’ daily life as invasive solutions are able to.

Dietrich et al.^21^ and Rokhmanova et al.^24^ were both able to demonstrate kinematic improvements while walking over ground and on stairs through the use of a simple ON-OFF stimulation paradigm that was remapped to the skin around the thigh in transtibial amputees. Pagel et al.^22^ and Rusaw et al.^23^ provided remapped feedback based on centre of pressure but presented contrasting results with Pagel demonstrating reduced postural control and reduced dependence on the prosthetic leg with feedback, and Rusaw demonstrating augmented standing balance with feedback. Interestingly, Rusaw employed modulated vibratory feedback^23^ as opposed to traditional ON-OFF stimulation, suggesting that intuitive stimulation modulation could be useful in bridging the gap between invasive and non-invasive solutions. Pan et al.^25^ also presented a more intuitive non-invasive feedback through the placement of a high density electrode grid behind the knee of transtibial amputees to directly target residual nerves where they are close to the skin surface and evoke somatotopic (spatially-matched) sensations. Nonetheless, the feasibility of this intervention must be proven in the functional walking applications, since the current results presented patients lying prone and immobile.

Transfemoral amputation is significantly more severe than transtibial because of the higher level of amputation and resulting loss of the knee joint^26^. In transfemoral amputees, Crea et al.^20^ and Martini et al.^27^ implemented unilateral and bilateral vibrotactile feedback systems respectively that provided time-discrete feedback based on gait events like toe-offs and heel-strikes. These feedback systems both demonstrated increased gait symmetry despite minimal sensory encoding schemes and testing in few walking conditions. The significance of these results for transfemoral amputees is underlined by the fact that sensory restoration could be even more important for this specific population due to their higher rates of disability and mortality^28–30^. Indeed, the loss of the knee and ankle together with a challenging residual limb cause additional problems in balance^31^ and gait symmetry^32^. In particular, the missing information from the knee flexion angle causes an increased risk of falls^3,32,33^, posing huge challenges for this population. Therefore, the possibility of providing health benefits explored by other studies, such as metabolic consumption^34^ and confidence^35^, is crucial for the transfemoral amputee population.

The non-invasive approaches in transfemoral amputees presented above are much less physiologically plausible than the invasive ones. Although they can provide real-time sensory feedback, they are lacking in their ability to provide somatotopic, direct pressure information as well as knee angle or other proprioceptive information that can currently be provided by invasive solutions.

This study explores how a non-invasive, multimodal system compares with analogous invasive approaches in transfemoral amputees. Here we present a functionally and cognitively comprehensive set of experiment results in two, functional classification level (K-Level) and age matched, transfemoral amputees and directly compare them with the findings of invasive approaches.

## Results

A non-invasive (NI) system^36^ providing touch and joint knee angle feedback was developed and tested in two transfemoral amputees (NI1 and NI2). Prior to functional testing, sensation characterization was performed to determine the location and quality of the perceived stimulation and the appropriate current and pulsewidth values for different intensity levels. Subsequently, to fully assess this technology, the subjects were asked to perform a variety of functional and cognitive tasks. These results were then compared with those obtained from two functionality level, age, and gender matched subjects provided with intraneural (I) sensory feedback (I1 and I2) during the same tasks.

### Electro-cutaneous (NI) stimulation provided feedback remapped to the thigh

**Figure 1:**
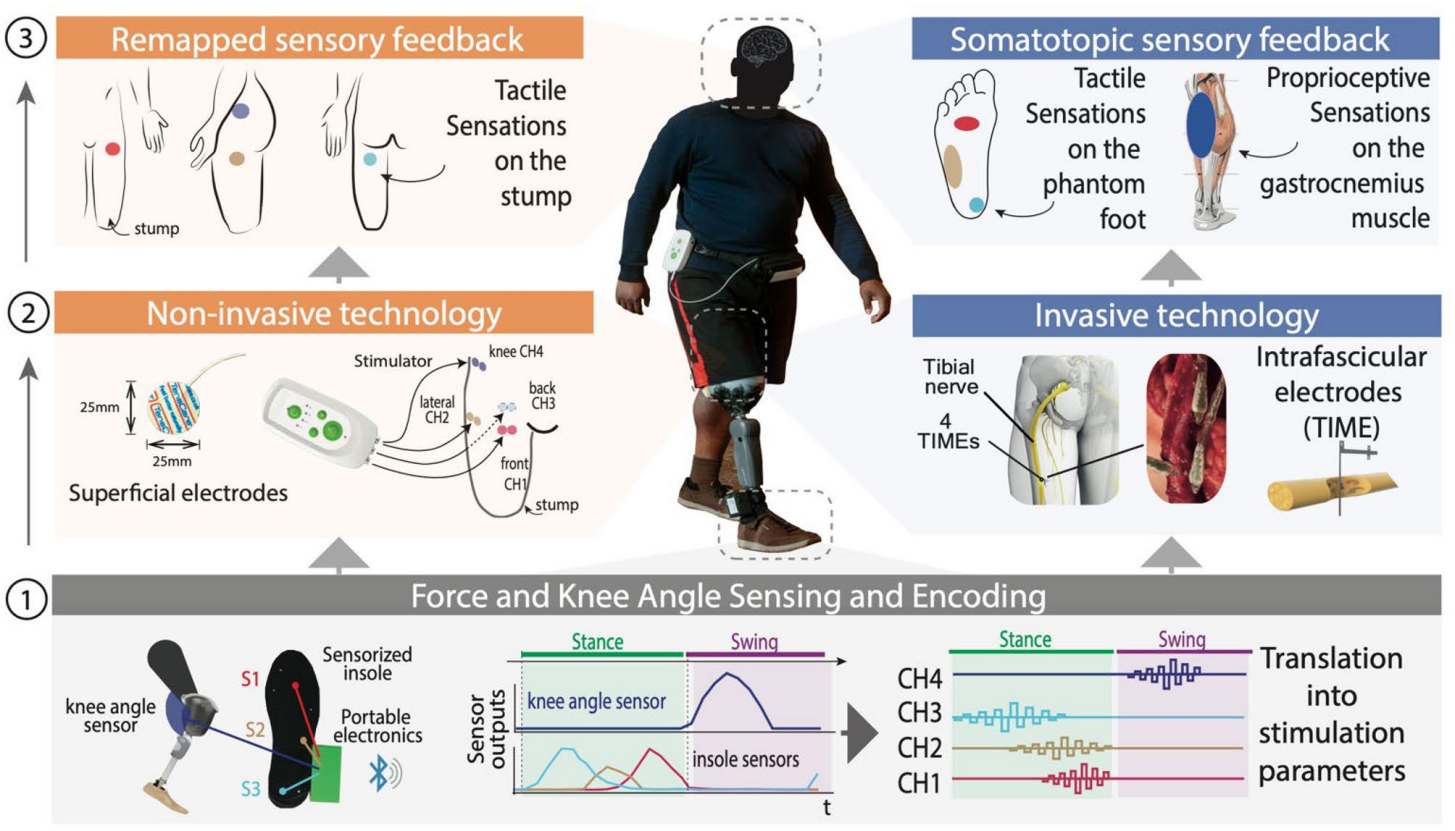
Non-invasive (NI) and Invasive (I) Sensory Feedback Systems. Real-time electro-cutaneous sensory feedback system composed of: (1) wearable sensors capturing in real-time the force exerted by the subject under the prosthetic foot and the knee flexion/extension of the prosthetic leg. Signals are encoded as stimulation by a microprocessor; (2) NI: surface electrodes placed on the stump and I: electrodes implanted tranversally through the tibial nerve^12^; (3) evoked sensation locations in both the NI and I case.

**Figure 2:**
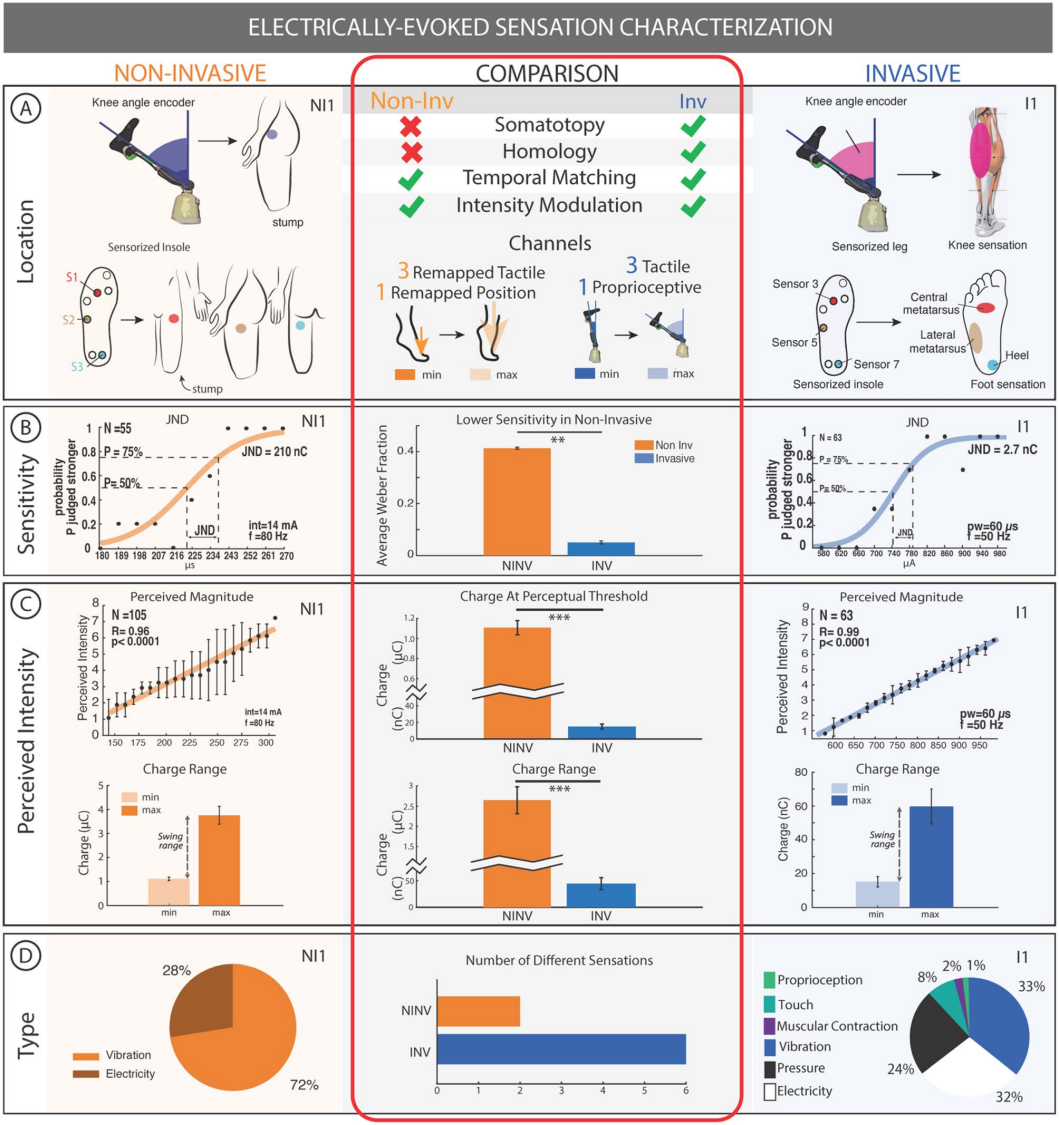
Electrically evoked sensation characterization. (**A**) Sensory feedback is mapped to the thigh with (NI1) and the foot sole + gastrocnemius muscle (I1) perceived magnitude. (**B**) The sensitivity of both NI1 and I1 is shown through their just noticeable difference (JND). (**C**) In both NI1 and I1, the perceived intensity increases with charge injected. The charge range is on the order of µC and nC for NI and I respectively. (**D**) Type of sensation and proportion of natural sensations for NI1 and I1 are presented in pie charts. All error bars indicate standard error. *p < 0.05, **p < 0.01, ***p < 0.001. Further results for NI2 and I2 are presented in Supplementary Figure S2A. The right column in panels A, B, C, and D are taken with permission from Preatoni et al.^5^.

As shown in Fig. 1 pressure values from the front, side, and heel of the insole were mapped with a proportional electrical stimulation to the front, side and back of the thigh while the knee angle was mapped to a position above the hip. This contrasts with the invasive approach that maps the front, side and heel of the insole to the central metatarsus, lateral metatarsus and heel respectively, with the knee angle being mapped to the gastrocnemius muscle^11,12^. In both NI and I cases, the area of sensation increases with force intensity and in the NI case it also increases with knee angle flexion (Fig. 2A). Details of perceived sensation location for each NI subject can be found in Fig. S1.

### Electro-cutaneous stimulation is less sensitive and required higher charge and charge range to achieve similar perceived intensity to intraneural stimulation

Sensation calibration was performed prior to functional testing to determine a specific perceivable injected charge difference for each subject so that intensity levels of stimulus could be encoded. For NI1, the perceived intensity was shown to increase with charge injected and the just noticeable difference (JND) of charge was 210 nC with a reference value of 3.15µC. For I1, the perceived intensity was also shown to increase with charge and the JND was 2.7nC with a reference of 46.8nC (Fig. 2B,C). The difference in JND and chosen reference value between NI1 and I1 is two orders of magnitude. The differences in average Weber fraction (JND/reference)^37^ found for each channel using JND were 0.41 ± 0.01, 0.41 ± 0.01 and 0.43 ± 0.02 in NI1 for the front, lateral and back channels respectively and 0.06 ± 0, 0.06 ± 0 and 0.04 ± 0 in I1 for the front, lateral and back channels respectively (Fig. 2B).

The charge injected at the perceptual threshold over all channels was significantly higher (Wilcoxon rank sum; p < 0.001) in NI subjects (1.107 ± 0.070 µC) compared to I subjects (15.1 ± 3.1 nC). The comfortable charge range over all channels was also significantly higher (Wilcoxon rank sum; p < 0.001) in NI subjects (2.649 ± 0.333 µC) compared to I subjects (44.6 ± 11.3 nC). The average Weber fraction found over all channels was significantly higher (Wilcoxon rank sum; p < 0.01) in NI subjects (0.4131 ± 0.0039) compared to I subjects (0.0506 ± 0.0060) (Fig. 2C).

The charge density injected through non-invasive electrical stimulation was from 0.226 ± 0.014 µC/cm^2^ to 0.7765 ± 0.076 µC/cm^2^ and in the invasive case was from 300.9 ± 61.1 µC/cm^2^ to 1187.9 ± 205.0 µC/cm^2^, both in the safe range of stimulation^38,39^ (Fig. S2B).

### Electro-cutaneous stimulation produced comfortable, but less rich sensory feedback than intraneural

The types of sensations reported by non-invasive subjects NI1 and NI2 were 72% vibration, 28% electricity for NI1 and 96% vibration, 4% tingling for NI2. The types of sensations reported by invasive subjects I1 and I2 were 33% vibration, 1% proprioception, 8% touch, 2% muscular contraction, 24% pressure and 32% electricity for I1 and 21% vibration, 29% pulsation, 2% warm, 22% tingling, 6% pressure and 20% electricity for I2 (Fig. 2D, Fig S2A). In NI subjects, there were an average of 2 different types of sensations elicited and in I subjects there were 6 different sensations on average. (Fig. 2D, Fig S2A)

### Fewer positions with higher accuracy over foot sole and knee closures could be recognized with electro-cutaneous stimulation

**Figure 3:**
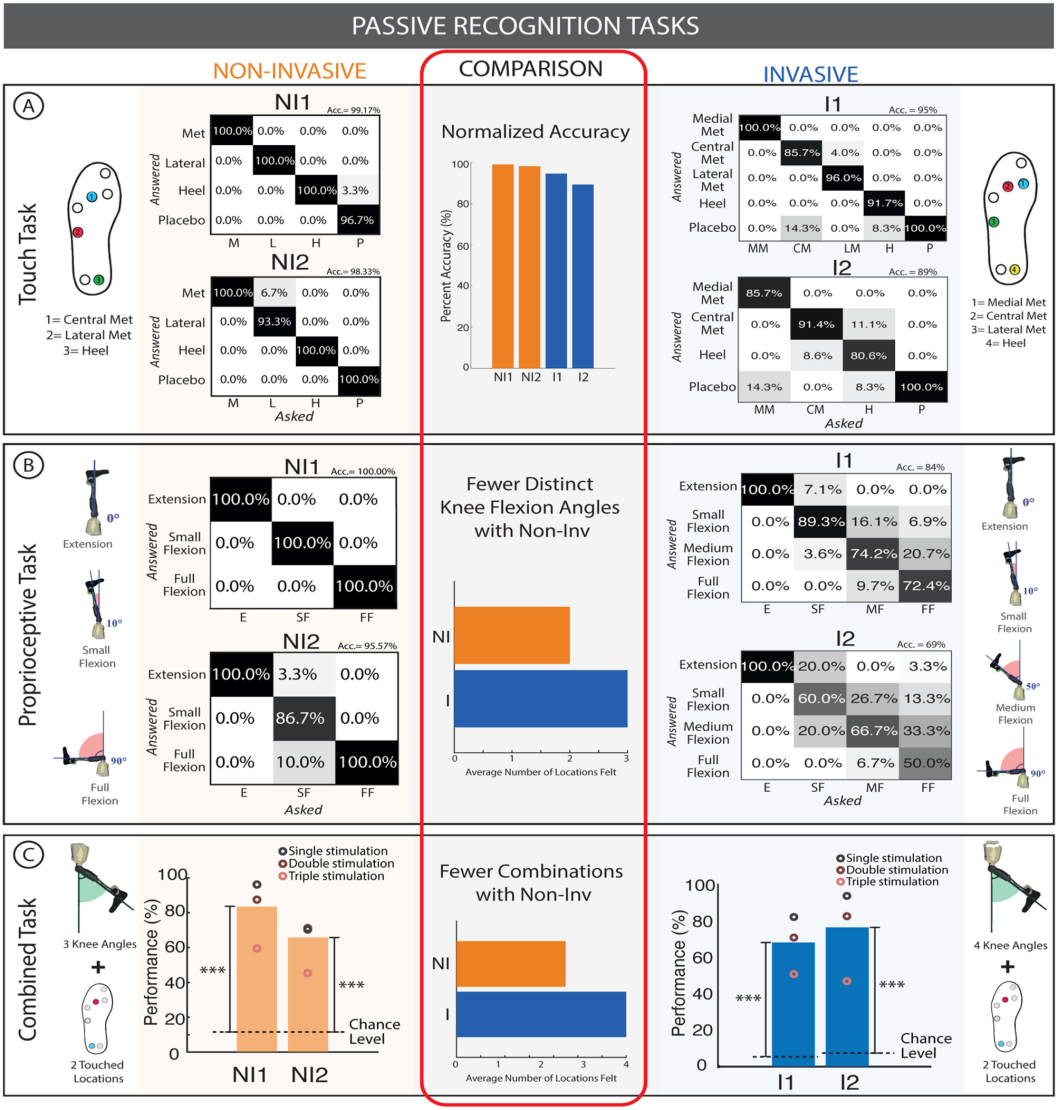
Passive recognition tasks. (**A**) Touch task results for three locations (NI/I) and four locations (I) as well as the normalized accuracy of each subject are shown. (**B**) Proprioceptive task results for two flexion angles (NI) and three flexion angles (I) are shown. (**C**) Combined touch and proprioceptive task results for a variety of locations and flexion angles are shown. All these results are compared in terms of number of distinguishable levels. A Fisher test was used to compare the results of the individual and combined tasks with their chance levels. ***p < 0.001.

After the sensation characterization process, the subjects (NI1 and NI2) performed passive recognition tasks^12^ similar to those performed in invasive (I1 and I2) subjects to evaluate their ability to exploit the remapped sensory feedback to accomplish sensory recognition tasks without visual feedback (Fig. 3). In this task, subjects were blindfolded while different points on their sensorized prostheses were touched (touch feedback) or their prosthetic knee was flexed (proprioceptive feedback). They were then asked to respond which part of the foot was touched or how flexed their knee was. This was done with touch feedback only, proprioceptive feedback only, and combined touch and proprioceptive feedback.

In the localized touch recognition task, NI1 correctly identified all the active stimulation (Front, Side, Heel and Placebo conditions) with an overall accuracy of 99.2% (N = 30 per condition). NI2 had an overall accuracy of 98.3% (N = 30 per condition). This is comparable in performance to the results presented by Petrini et al.^12^ with invasive sensory restoration where invasive subject I1 had an overall accuracy of 92.4% and I2 had an overall accuracy of 95%^12^. The normalized accuracy ((accuracy – probability)/(100 – probability) × 100)) was found to be 99.17% for NI1, 98.32% for NI2, 94.67% for I1, and 89.40% for I2 (Fig. 3A) Three spatially separated locations were chosen based on multiple previous invasive studies^5,11,12,35^. Additionally, Crea et al^20,40^ successfully implemented three spatially separated vibrotactile stimulators in transfemoral amputees. Moreover, the low tactile acuity of the thigh relative to the foot sole^41^ makes it difficult to remap foot sole information to the thigh. The successful discriminability of these remapped locations verifies the successful intuitive encoding of the foot sole locations related to the stance phase periods of gait.

In the flexion level recognition task (i.e. the knee angle), NI1 had an overall accuracy of 100% (for Extension, Small flexion, Full Flexion) (N = 30 per condition). NI2 had an overall accuracy of 95.6% (N = 30 per condition). Initially, 4 levels were tried with non-invasive subjects, but they could not recognize the 4th level, so the number of levels was decreased to 3 to improve performance. Comparatively, I1 had an overall accuracy of 77.7%, and I2 had an overall accuracy of 83.7%. Both invasive study subjects could identify 4 different levels of flexion as opposed to 3 (Fig. 3B)^12^.

In the combined touch and flexion level recognition task, NI1 had an overall accuracy of 84.4% (N = 135), where 63.3% was the accuracy for 3 distinct stimulations (triple stimulation—front + heel + knee) active at the same time (N = 30), 88% was the accuracy with two stimulation channels (double stimulation) (N = 75), and 96.7% was the accuracy with only one channel active (single stimulation) (N = 30). NI2 on the other hand had a lower accuracy, with 65.7% accuracy overall (N = 135), with 45.8% for the triple stimulation (N = 30), 71.7% (N = 75) for the double stimulation, and 70.8% (N = 30) for the single stimulation. In all cases, the accuracy levels were higher than random chance level (of 11%). In the invasive study, I1 had an overall accuracy of 67.4% (N = 308) where 50.6% (N = 85), 70.6% (N = 146), and 81.8% (N = 77) were the accuracies for triple, double, and single stimulation respectively and I2 had an overall accuracy of 76.2% where 46.7% (N = 45), 82.2% (N = 90), and 93.3% (N = 45) were the accuracies for triple, double, and single stimulation respectively^12^. Both NI1 and NI2 distinguished nine different combinations of stimulus and I1 and I2 distinguished 16 and 12 combinations respectively. There was a high variation in the number of combinations of stimulus in the invasive study, but overall, more combinations were presented in the invasive task when compared to non-invasive (Fig. 3C).

The touch and proprioceptive discrimination accuracy were compared to their respective control conditions^12^ (Fig. S3). Both NI and I subjects in both the non-invasive and invasive sensory feedback conditions had higher accuracies with respect to the control conditions when sensory feedback was off (N = 60). The results for these control conditions can be found in Fig. S3 for both non-invasive and invasive sensory feedback.

### Subjects notice no change in the weight and embodiment of the prosthesis with non-invasive sensory feedback

**Figure 4:**
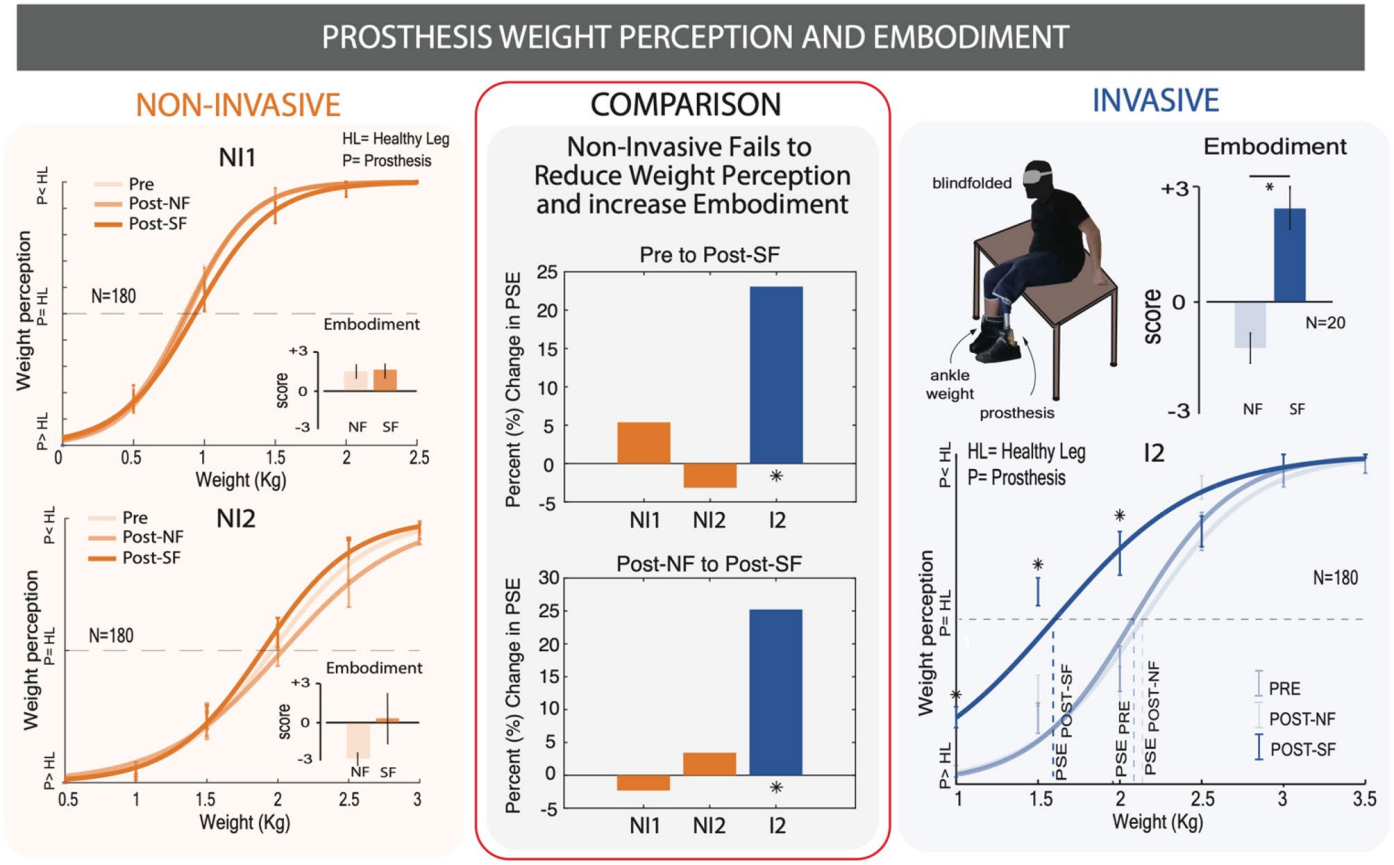
Weight perception and embodiment. subjects performed a weight perception discrimination task before and after a sensory feedback task and no feedback task. Psychometric curves representing their perceived prosthesis weight are presented for NI subjects and I subjects. Statistical significance for specific test weights is marked with *p < 0.05. Self-reported embodiment on a −3 to 3 Likert scale are also shown as graph insets. A comparison between NI and I performance is drawn. The right column is taken with permission from Preatoni et al.^5^.

Weight perception was evaluated using a motor task as in Preatoni et al.^5^. In this task, subjects completed a motor task with or without sensory feedback. Before (PRE) and after (POST-SF, POST-NF) this motor task, their weight perception was evaluated by having them sit on the edge of a table with both the healthy and prosthetic leg hanging off the edge, not touching the ground. Subjects were blindfolded while six differing weights were strapped to their healthy leg and they were asked to respond with which leg felt heavier. The answers of the subjects in each condition were fitted with a psychometric function (logit) with a linear method. Then, the point of subjective equality (PSE) was calculated by finding the point at which the subjects perceived the weight of their healthy limb and their prosthesis to be the same (i.e., weight that had to be added to perceive the limb as equally heavy). For I2 the PSE was found to be 2.08 kg, 2.14 kg, and 1.6 kg in the PRE, POST-NF, and POST-SF conditions respectively^5^. For NI 1 it was 0.93 kg, 0.86 kg, and 0.88 kg in the PRE, POST-NF, and POST-SF conditions respectively. For NI 2 it was 1.9 kg, 2.03 kg, and 1.96 kg in the PRE, POST-NF, and POST-SF conditions respectively. Statistically significant results are only found in the case of invasive sensory feedback: the ratings in the POST-SF condition were significantly different for the first three ankle weights, yielding a reduction of 23% (0.48 kg) in the POST-SF PSE compared to the baseline (Fig. 4). In NI subjects, none of the different weights were perceived differently in the POST-SF condition compared to the PRE and POST-NF conditions. This means that the PSE in the non-invasive condition did not change with or without SF. (Fig. 4)

This result is confirmed also by the occurrence of each rating (prosthesis heavier, equal weight, and healthy leg heavier) (Fig. S4).

The embodiment, or ownership of the prosthetic was evaluated with a standard questionnaire^5,12,42^ after each motor task. The prosthesis embodiment was evaluated to quantify how much the subject perceived the prosthetic device with the sensory feedback as part of their own body^5,12,43^. The results of the embodiment questionnaire showed that I2 had statistically significant (Friedman test; p = 0.01) scores of 1.2 ± 0.39 after the NF condition and 2.43 ± 0.54 after the SF condition^5^. Neither NI1 or NI2 had statistically significant scores with NI1 having scores of 1.679 ± 0.643 after NF and 1.726 ± 0.665 after SF, and NI2 having scores of −2.822 ± 0.4127 after NF and 0.7444 ± 2.457 after SF (Fig. 4).

### Partial improvement in dual task performance with non-invasive sensory feedback

Subjects were asked to perform a mental workload (MW) task involving spelling a word backwards in their mother tongue while walking forward for 5 m. The spelling accuracy and their speed while walking were evaluated.

**Figure 5:**
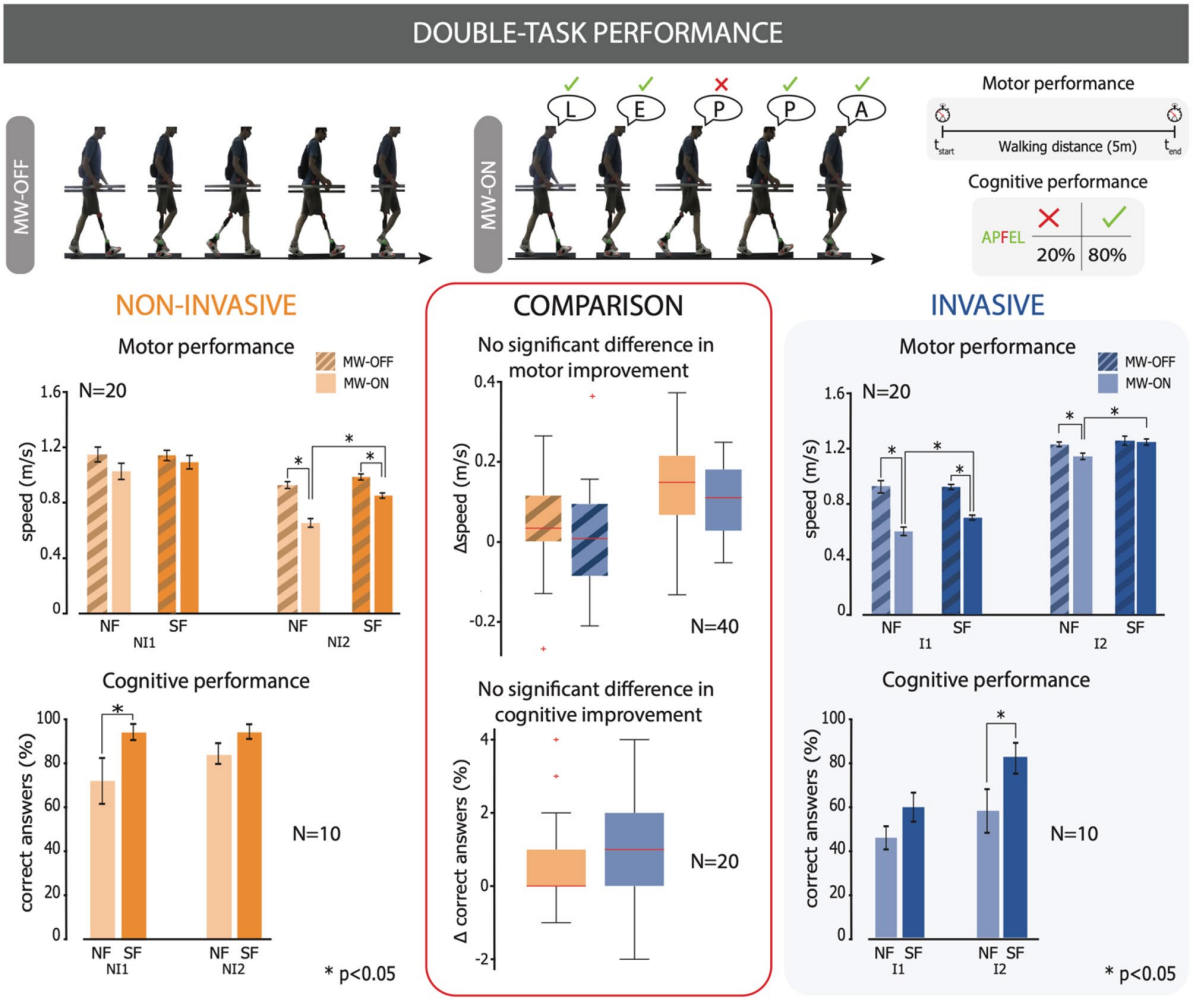
Double task performance. Subjects performed a simultaneous cognitive (spelling words backwards) and motor (walking) task with and without sensory feedback. Their cognitive performance was evaluated through the number of correct letters and their motor performance was evaluated through walking speed. Comparisons between the cognitive and motor improvements of NI and I subjects was also drawn by grouping the paired data together. Statistical significance is marked with *p < 0.05, **p < 0.01, ***p < 0.001. Parts of the right column are taken with permission from Preatoni et al.^5^.

NI1 did not have a significant change in walking velocity with SF (1.138 ± 0.037 m/s) compared to NF (1.132 ± 0.053 m/s) in the MW-OFF condition and did not have a significantly increased walking velocity with SF (1.093 ± 0.049 m/s) compared to NF (1.026 ± 0.059 m/s) in the MW-ON condition. NI2 did not have a significant change in walking velocity with SF (0.995 ± 0.022 m/s) compared to NF (0.925 ± 0.025 m/s) in the MW-OFF condition, but did have a significantly increased walking velocity (Wilcoxon signed rank test, p < 0.01) with SF (0.859 ± 0.019 m/s) compared to NF (0.648 ± 0.031 m/s) in the MW-ON condition. I1 did not have a significant change in walking velocity with SF (0.925 ± 0.018 m/s) compared to NF (0.931 ± 0.045 m/s) in the MW-OFF condition, but did have a significantly increased walking velocity (Wilcoxon signed rank test, p < 0.05) with SF (0.703 ± 0.017 m/s) compared to NF (0.604 ± 0.030 m/s) in the MW-ON conditions. I2 did not have a significant change in walking velocity with SF (1.289 ± 0.033 m/s) compared to NF (1.252 ± 0.017 m/s) in the MW-OFF condition, but did have a significantly increased walking velocity (Wilcoxon signed rank test, p < 0.01) with SF (1.280 ± 0.023 m/s) compared to NF (1.173 ± 0.024 m/s) in the MW-ON condition. (Fig. 5).

NI1 identified 36 letters correctly and 14 incorrectly with NF and 47 letters correctly and 3 incorrectly with SF (Fisher test, p < 0.05). NI2 identified 42 letters correctly and 8 incorrectly with NF and 47 letters correctly and 3 incorrectly with SF. I2 identified 23 letters correctly and 27 incorrectly with NF and 30 letters correctly and 20 incorrectly with SF. I3 identified 29 letters correctly and 21 incorrectly with NF and 41 letters correctly and 9 incorrectly with SF (Fisher test, p < 0.05) (Fig. 5).

The difference in the performance with and without sensory feedback between NI and I subjects were directly compared. The improvement in velocity with SF when performing a memory task was 0.139 ± 0.028 m/s in NI subjects and 0.103 ± 0.021 m/s in I subjects. The difference in velocity between SF and NF when not performing a memory task was 0.038 ± 0.025 m/s in NI subjects and 0.015 ± 0.030 m/s in I subjects. The improvement in cognitive task performance with SF was also computed and found to be 0.85 ± 0.31 answers in NI subjects and 0.95 ± 0.37 answers in I subjects. None of these three comparisons was found to be statistically different (Fig. 5).

### Improvement in the performance of daily life tasks

Participant NI1 competed as a part of team NeuroLegs at Cybathlon 2020 Global Edition, a competition for people with physical disabilities to compete against each other in daily life tasks with the assistance of state-of-the-art technology^44^.

Data collected during training sessions showed that, with SF and NF respectively, the points per second (PPS) achieved was 0.9797 and 0.9245 PPS for the precision task, 0.4195 and 0.3454 PPS for the Dynamic Balance Task, 0.3794 and 0.3791 PPS for the Mobility Task, 0.8517 and 0.7536 PPS for the Stability Task, 1.2567 and 1.2199 PPS for the Static Balance Task, and 0.5187 and 0.5462 PPS for the Agility Task (Fig. S5).

## Discussion

The results of an electro-cutaneous, non-invasive feedback system tested in two transfemoral amputees were compared to those of an analogous intraneural system in two, separate, functionality level, age, and gender matched transfemoral amputees. With this non-invasive system and the calibration method that accompanies it, both subjects were able to identify distinct locations and closures of the leg, or their combinations. Yet the number of recognized positions and combinations of position/movement, were smaller with respect to the invasive solution, probably due to the lower intuitiveness of its interpretation by the CNS compared to the neuro-integrated case. This is consistent with other non-invasive solutions that stimulate distinct locations^20,21,23,24^, but is unique in that it is the only non-invasive system where transfemoral amputees can recognize and exploit different stimulation levels.

Although it appears that fewer levels were tested in NI subjects than I subjects (Fig. 3B), NI subjects were first tested with the same number of levels as I subjects if their calculated Weber Fraction and perceptual range indicated it was possible (i.e. if the (perceptual threshold) × (Weber Fraction)^4^ was less than the maximum comfortable threshold). They were only tested to confirm the recognition of fewer levels if they failed this initial task (Fig. S6). The lack of accuracy in level calibration may come from an imprecise JND estimation. The estimation of JND considered each subject’s entire perceptual range and used the same number of tested values and repetitions spread out through this perceptual range in every subject. This meant that for more sensitive subjects, such as NI1, the transient range of the psychometric curve used to estimate JND contained few tested values and could not be well fit. Considering personalized JND calibration ranges could improve JND estimation and therefore stimulation level calibration in the future.

The non-invasive (NI) system was able to improve multitasking in a manner similar to intraneural sensory feedback. During the dual task, both the NI and I systems allowed the subjects to improve their walking speed or decrease their mental workload. Notably, there was no difference in the amount of improvement given by the invasive solution compared to the non-invasive. However, in one invasive case there was an improvement of both walking speed and spelling accuracy. On the other hand, the non-invasive approach allowed one subject to increase walking speed but not spelling accuracy, while for the other subject we saw benefits only in spelling. These findings can be interpreted as a partial cognitive integration of the non-invasive sensory feedback^5,45^. It is possible that the different types of SF we provided impacted the integration of the prosthesis in subjects’ body schema. The intraneural SF allowed both somatotopic and homologous sensations, immediately and without any training, since it was using the pathways devoted to prior function, something impossible with the remapped SF used in the non-invasive system. The non-physiological signals elicited had to be learned by the central nervous system^46^ in order to link the stimulation on the thigh with specific prosthetic limb states. This cognitive process may require a higher cognitive burden and get in the way of optimally integrating the artificial SF^7,47^. This is consistent with how non-invasive interventions typically result in a higher cognitive load as they are non-homologous and non-somatotopic.

In other regards, the non-invasive electro-cutaneous stimulation performed worse than intraneural stimulation. It required higher charge therefore increasing battery demands. It also supported charge densities lower than invasive. It evoked fewer sensations naturally associated with walking (i.e. homologous sensations such as touch and pressure^9,48^) and did not improve prosthesis weight perception as intraneural sensory feedback did likely due to its non-invasive, remapped nature. This is consistent with other non-invasive approaches^20,21,23^ and naturally follows from its inability to evoke somatotopic, homologous sensations as discussed above. A somatotopic non-invasive feedback may be able to encode more information and improve the intuitiveness of the sensory feedback since it would bypass the remapping process that results in fewer evoked sensations and a reduced number of distinguishable sensation levels. This could be improved in future iterations of the device if nerves near the surface of the skin on the stump can be stimulated non-invasively. This is quite difficult to achieve in transfemoral amputees where the tibial nerve is far from the surface of the stump, but shows promise in transtibial amputees where sensory nerves can be stimulated non-invasively from behind the knee^25^.

Another possible solution to obtain an optimal integration with a remapped sensation, could be training the subjects with the non-invasive SF, and possibly virtual reality, within a multimodal platform^7^. To efficiently use the information provided by the sensory substitution, the brain has to create new, unused neural pathways, which requires training^46^. Humans develop their senses over years and the ability to use a new SF might need more time to be naturally integrated, which eventually may translate into both motor and cognitive benefits. This hypothesis is supported by the lack of significance in embodiment scores and might be a further reason behind the differences we saw in the weight perception task, where the NI approach failed in reducing the perception of the weight of the prosthesis. Nevertheless, the significance of even partial cognitive integration and the improved dual task performance that accompanies it is highlighted through the importance of daily life tasks, which have been shown to require multitasking^49^. The ability of the NI system to augment multitasking is validated in its performance at Cybathlon, a rehabilitation engineering competition involving the performance of daily life tasks. An example of these tasks is moving flatware from a low coffee table to a high dining table, a task that requires both hand-eye coordination, and lower limb stability and balance. This system was showcased as part of team NeuroLegs, and helped the pilot of the leg come in second place^44^.

Although this non-invasive system naturally does not match the level of performance or provide the same diverse array of benefits as invasive approaches, it solves the problem of surgery for individuals who are simply looking to improve their ease of performing daily life tasks. Previous non-invasive studies in transfemoral amputees have demonstrated some improvements in kinematic function but did not explore its significance for the cognitive integration^20,27^. The results presented here complement these findings through demonstrating that a non-invasive device reduces cognitive load in a manner similar to more targeted implantable solutions.

The preliminary evidence obtained through this two-participant study highlights the main drawback of non-invasive approaches: their remapped nature. As seen through conducting these experiments and experiencing the tedious calibration highlighted in other publications^36^, one of the most useful next steps in non-invasive stimulation technology would be the development of a stimulation method that can directly target the nerve. This would open the door to other applications and possible benefits, such as reducing phantom limb pain^50^. Particularly, previous studies have shown that directly stimulating the nerve can drastically decrease pain levels^11^. This is based on the Gate Control Theory, which suggests that by stimulating large diameter AB fibers it is possible to decrease the activity of nociceptive fibers through an inhibitory pathway in the spinal cord. Clearly this is impossible with the current technology that is completely remapped and does not directly target the nerves.

## Conclusion

The results shown in this paper suggest that only few improvements can be made with non-invasive technology. However, they take important steps towards understanding how prosthesis embodiment can be achieved with non-invasive solutions, and lead to a broader set of benefits for individuals who want to avoid a surgery^51^. This paper also provides a valuable direct comparison between invasive and non-invasive technology. When considering a future with wide-spread use of neural prosthesis^52^, non-invasive systems show promise as highly accessible solutions that can improve some aspects of the daily life of users. As in other areas of human technology, different tailored solutions would exist for different patient expectations where non-invasive systems could represent a valuable “base-model” alternative to more sophisticated invasive solutions, that come with the cost of surgery.

## Methods

### Participants

Two subjects with traumatic unilateral lower-limb amputation at the transfemoral level participated in this study (Table 1). Both amputees were male and were respectively 54 and 31 years old with a K-Level of 4. The comparative invasive subjects were also both male and 49 and 35 years old respectively with a K-Level of 4^11^. The experiments were approved by the ETH Zurich’s ethics commission (EK 2019-N-97, Approved: 27/11/2019). The trial was registered with ClinicalTrial.gov (NCT04217005, First Posted: 03/01/2020). The experiments were performed in accordance with the proposal approved by the ETH Zurich’s ethics commission and in accordance with the Declaration of Helsinki. All subjects read and signed the informed consent including the use of identifiable images in an online open-access publication.

**Table 1:** Patients’ demographics.

	Sex	Age	Amputation level and side	Amputation cause	Year of amputation	K-level	Prosthesis	Frequency of use	Phantom limb pain or other underlying conditions
NI1	M	54	Distal two-thirds of the left leg (transfemoral)	Trauma	2013	4	Ottobock GENIUM X3 and Ottobock VARI-FLEX LP	Daily	No
NI2	M	31	Distal two-thirds of the left leg (transfemoral)	Trauma	2018	4	Ottobock R and Össur PRO_FLEX XC foot	Daily	No

### Electro-cutaneous stimulation and pressure sensor calibration

First, in both NI and I subjects sensation and force calibration was performed, then passive recognition and control tests, and last functional tests in a randomized order. None of the participants received any systematic or prolonged training prior to the tests.

The electro-cutaneous stimulation was delivered using the system described by Basla et al.^36^ as a train of balanced, cathodic-first pulses of current. The frequency of the trains was maintained at 80 Hz, and the current was fixed at a specific level determined by the calibration process per-channel per-session. The location of the 3 stimulation channels was chosen to intuitively encode information from different parts of stance phase^40^. The sensation locations and types for the electrodes of the two subjects were reported in Fig. S1.

The variation of force exerted was encoded as an increase of pulse width to increase the charge injected through each electro-cutaneous pulse. The size of each increment was determined through the finding of a just noticeable difference identified during the advanced optimal calibration process. This calibration employed a forced choice task where subjects were presented for 110 times with the reference stimulus and a test stimulus then asked to judge which was stronger. Each pair of test and reference stimuli was repeated 10 times, so that the psychometric curve from which JND is extracted was fit to points representing an average of 10 repetitions^7^. This allowed us to create personalized, discriminable steps between the perceivable threshold and the maximum comfortable threshold.

The stimulations were meant to be comfortable and localized to just under the electrode pairs, activating only skin mechanoreceptors as opposed to somatotopic nerves as in invasive interventions^11,12^. Each subject was also asked to draw the location and size of the sensation as well as describe how it felt by picking from a list of descriptors ('Pressure', 'Twitch/Muscle Contraction', 'Tingling', 'Touch', 'Warm', 'Cold', 'Pain', 'Electricity', 'Pulsation', 'Vibration').

The insole forces were recorded during a force calibration session where each subject was asked to walk at a comfortable speed over level ground while wearing the sensorized insole. The maximum and minimum values were used to identify the useful dynamic sensor range, over which to map the stimulation range. The comfortable range of stimulation (between the perceptual threshold and maximum comfortable threshold) was unique to each subject and each pair of electrodes. The perceptual threshold was determined when the subject reported a 2/10 intensity and the maximum comfortable threshold was an 8/10 intensity^53^.

### Passive recognition tasks and control tests

As a further validation of the non-invasive sensory feedback, a series of passive recognition tasks were performed. In all the tasks with SF, the subjects were acoustically and visually isolated and had the prosthesis detached. The tasks were divided depending on the type of information conveyed: localized touch only, knee flexion only, or combined.

The first task was the recognition of *localized touch* only (Fig. 3). Each possibility, namely: (1) metatarsal (CH2, Met, frontal channel), (2) lateral (CH3, lateral channel), (3) heel (CH4, back channel) and (4) placebo, were tested in a random order. The investigator would press on the sensor of interest (or as in case of 4, not press at all), subsequently the subject was requested to answer by voice and show on his hand which channel he felt stimulated. A familiarization phase where the task was described to the subject (N = 5 per condition) was performed before the testing session.

The second task evaluated *proprioceptive feedback* only (Fig. 3). In this case, only the channel used to provide information about the knee flexion was used (Ch1). The flexion conditions were: (1) extension (no stimulation), (2) small flexion (low intensity stimulation) and (3) full flexion (high intensity stimulation). The order of the possible flexions was randomized and provided to the subject, which had to reply the level of flexion felt, in the same way as in the localized touch task.

The third task was the *combined touch and proprioceptive feedback* (Fig. 3). In this case, the subject was simultaneously exposed to two different types of sensory information: one related to foot touch, and one to knee flexion. The channels used in this case were the Metatarsal (CH2, on the front of the stump), Heel (CH4, on the back of the stump), and the one dedicated to knee flexion (CH1, on the iliac crest). The possible combinations of stimulation could either use one channel at the time (single stimulation), two (double stimulation), or three (triple stimulation), for a total of nine different conditions. Namely the combinations were: (1) Extended knee + Metatarsal; (2) Extended knee + Heel; (3) Small flexion + Metatarsal; (4) Small flexion + Heel; (5) Full Flexion + Metatarsal; (6) Full Flexion + Heel; (7) Extended knee + Metatarsal + Heel; (8) Small flexion + Metatarsal + Heel; (9) Full Flexion + Metatarsal + Heel. As in the other tasks, the order of stimulation was randomized, the subjects were blinded and acoustically isolated, and instructed to answer by showing numbers on their hand and using their voice.

In the *control tests*, (Fig. S3) the subject was acoustically and visually isolated while wearing the prosthesis exploiting only the residual haptic feedback provided by the stump-socket interaction. No electro-cutaneous stimulation was provided. They were sitting on a chair in a way to allow the flexion of the prosthetic knee and to allow the investigator to press underneath the prosthetic foot. Both the localized touch task and proprioceptive task were performed in the control condition.

### Prosthesis weight perception task

Prosthesis weight was measured by asking a subject to sit on the edge of an elevated surface and hang the lower part of their legs off the edge. The subject was then blindfolded and weights were attached to the ankle of the healthy leg. Subjects were asked to rate which leg felt heavier as the weigh was varied (Fig. 4)^5^. This weight perception task was performed at a baseline (PRE), after a 10-min overground walking task without sensory feedback (POST-NF), and after a 10-min overground walking task with sensory feedback (POST-SF). Six different ankle weights were tested 30 times in each condition per participant. Over all conditions, there were 180 repetitions split over 3 sessions, resulting in total of 540 weight measurements per participant. More details about this task can be found in Preatoni et al. 2021^5^.

### Dual task

The mental workload task (MW) was performed over a period of one week as in Preatoni et al.^5^, the dataset which we refer to. Subjects were first asked to walk 5m while timed (MW-OFF, baseline for the primary task). This task was repeated a total of 20 times (10 times SF and 10 times NF) in a random order. The walking speed of the subjects were calculated for each trial. Subsequently, subjects were asked to complete the same walking task while performing a dual task (MW-ON). Specifically, subjects were asked to spell a five-letter word which had not been previously presented backwards in their mother-tongue (Fig. 5). This task was repeated a total of 20 times (10 times SF and 10 times NF) in a random order. While the subject was performing the MW-ON condition, both the walking speed and the accuracy of the spelling (as % of correct letters) were recorded^5^.

### Cybathlon

The points per second (PPS) acquired for each completed Cybathlon daily life task was calculated during training sessions before the competitions. Participant NI1 ran the full course once with sensory feedback on (SF) and once with sensory feedback off (NF). Each daily life task was given a number of points by the competition designers^44^ based on its difficulty. Points were only awarded if the entire task was completed successfully.

$${PPS}_{Task}=\frac{{Points}_{Task}}{{Completion\_Time}_{Task}}$$

### Self-reported embodiment assessment

At the end of each motor task, participants were asked to complete an embodiment questionnaire^42^ (the same as in Petrini et al. 2019^8^ excluding vividness and prevalence questions). The data were acquired in SF and NF conditions in all the subjects.

### Data collection and statistical analysis

Data was analyzed using Matlab (R2019b, The Mathworks, Natick, MA, USA). Statistical analysis was performed using built-in Matlab functions. To test whether the collected data were normally distributed, a one-sample Kolmogorov–Smirnov test was performed. Normally distributed data was analyzed using the two-sample t-test. For the few non-normal datasets, the two-sided Wilcoxon rank sum test was used to evaluate statistical significance when comparing inter-subject (independent) and the Wilcoxon signed-rank test was used to compare intra-subjects (dependent). The Fisher test was used to compare percentage success rates in binary tests. Levels that were found to have a statistically significant difference with α = 0.05 and *p* < 0.05 were marked with an asterisk reported on the plots.

## Supplementary Information

Supplementary Information.

## Data Availability

The datasets generated during and/or analysed during the current study are available from the corresponding author on reasonable request.
