# Supplementary material for: Cognitive benefits of using non-invasive compared to implantable neural feedback
Source: Sci Rep. 2022 Oct 6;12:16696. doi: 10.1038/s41598-022-21057-y (PMC9537330; doi:10.1038/s41598-022-21057-y)
Supplement: Supplementary file 1 — Supplementary Information. [file 41598_2022_21057_MOESM1_ESM.pdf]

Supplementary Materials

Cognitive benefits of using non-invasive compared to implantable neural feedback

Lauren Chee[1,+], Giacomo Valle[1,+], Greta Preatoni[1], Chiara Basla[1], Michele Marazzi[1], and Stanisa Raspopovic[1,*]

[1] Laboratory for Neuroengineering, Department of Health Science and Technology, Institute for Robotics and Intelligent Systems, ETH Zürich, 8092 Zürich, Switzerland

[*]stanisa.raspopovic@hest.ethz.ch

[+]these authors contributed equally to this work

Tables

	Sum of Squared Residuals (SSE)	R-squared	Root Mean Square Error (RMSE)
NI1	0.139	0.9223	0.1243
NI2	0.8546	0.8384	0.135

Table S1. JNDs Goodness of Fit for NI subjects

	Stimulation Channel	Pulse-width (µs)					Amplitude (mA)
		L1	L2	L3	L4		
NI 1	Ch 1	136	198	278	391		7
	Ch 2	140	196	278	390		7
	Ch 3	134	190	267	374		11
	Ch 4	134	191	278	390		10

Table S2. Stimulation Pulse-Widths and Amplitudes for Subject NI1.

Figures

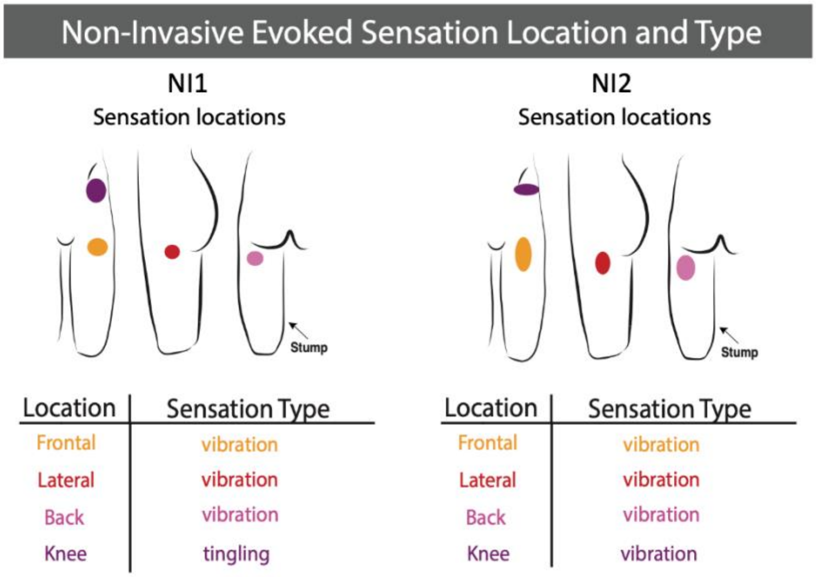

Figure S1 Non-Invasive Sensation Characterization. Perceived sensation locations and types in NI subjects.

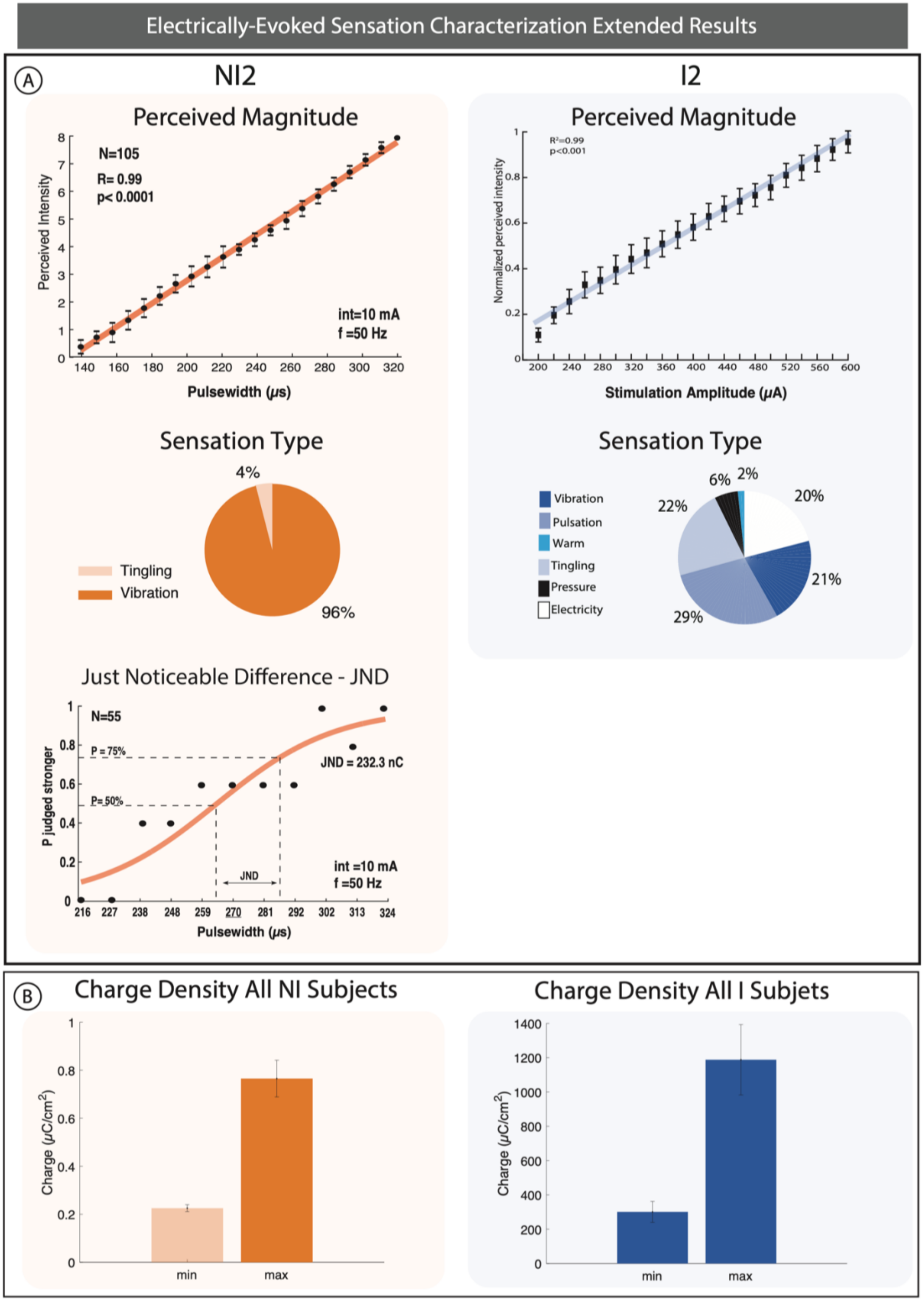

Figure S2 Electrically-Evoked Sensation Characterization Extended results. (A) Electrical charge sensitivity, perceived intensity of charge, and sensation type for NI2 and I2. (B) Charge Density in all NI and I subjects.

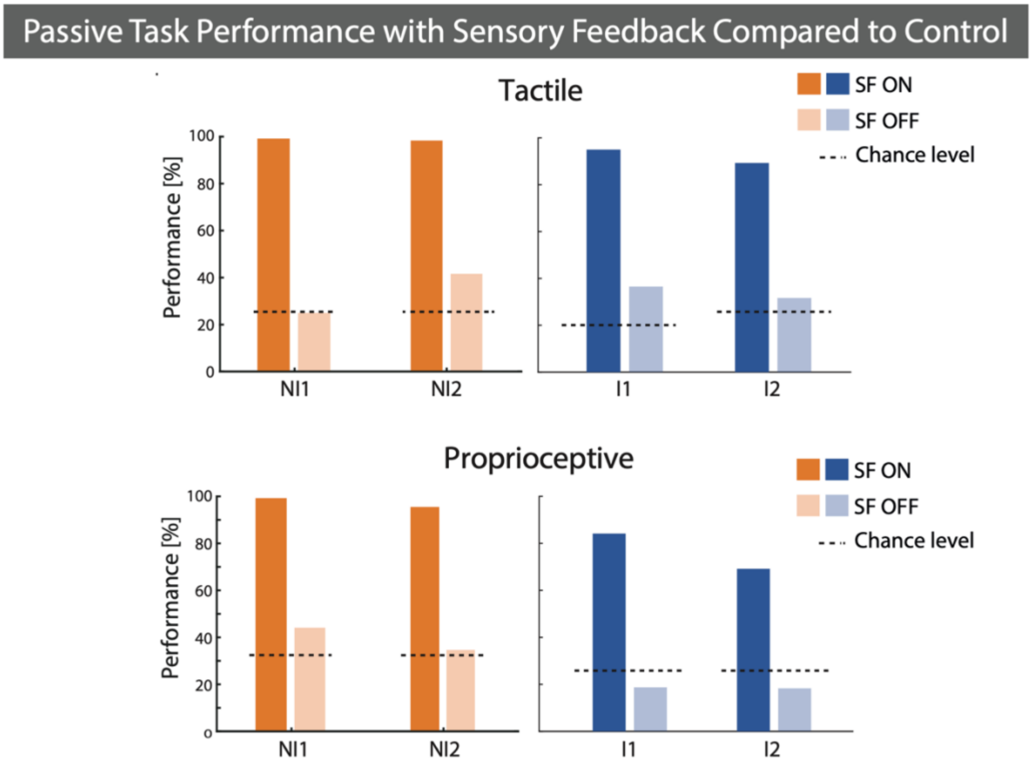

Figure S3. Passive Task Control Conditions. Correct passive identification of touched location or flexion level for Non-invasive subjects NI1, NI2 and invasive subjects I1, I2.

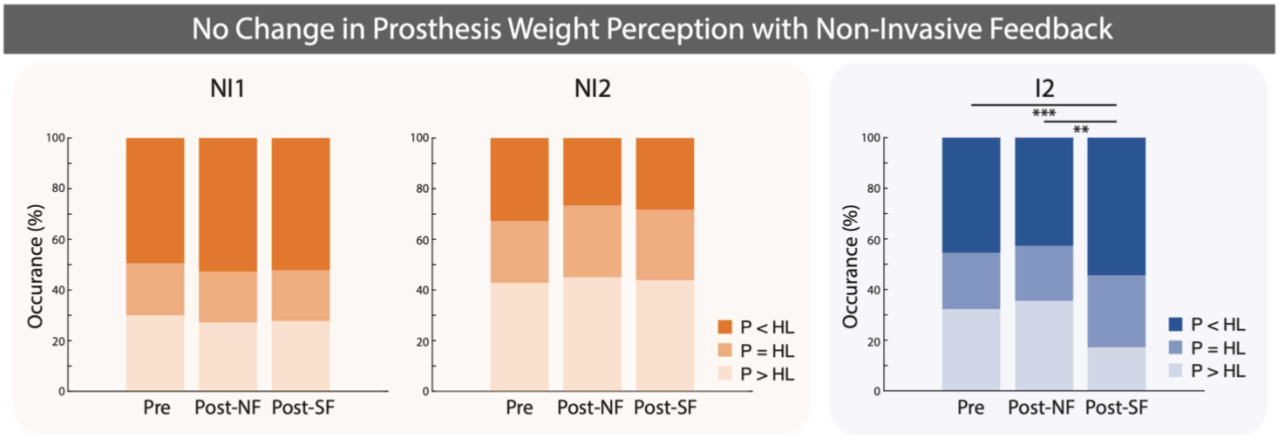

Figure S4. Prosthesis Weight Perception Relative to Healthy Leg. Occurrence of response to the question "Which leg is heavier". P = prosthetic, HL = healthy leg. Statistical significance is marked with *p<0.05, **p<0.01, ***p<0.001.

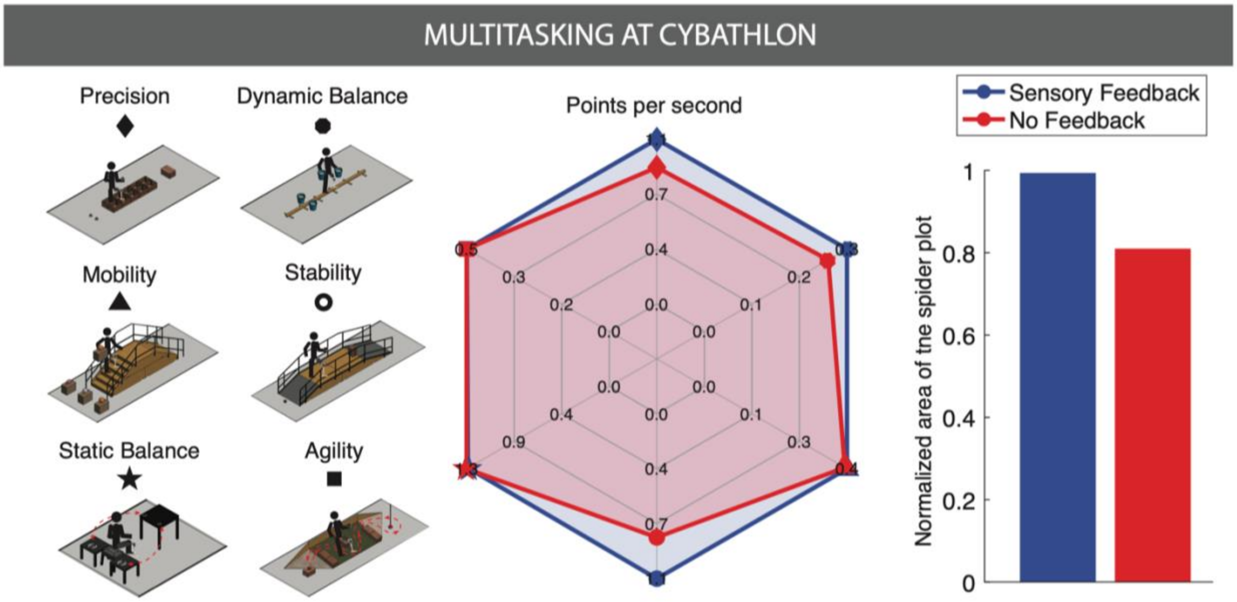

Figure S5. Daily Life Task Performance at Cybathlon. Points per second achieved in each of the daily life tasks at Cybathlon are plotted in a spider plot and compared with and without sensory feedback for NI1

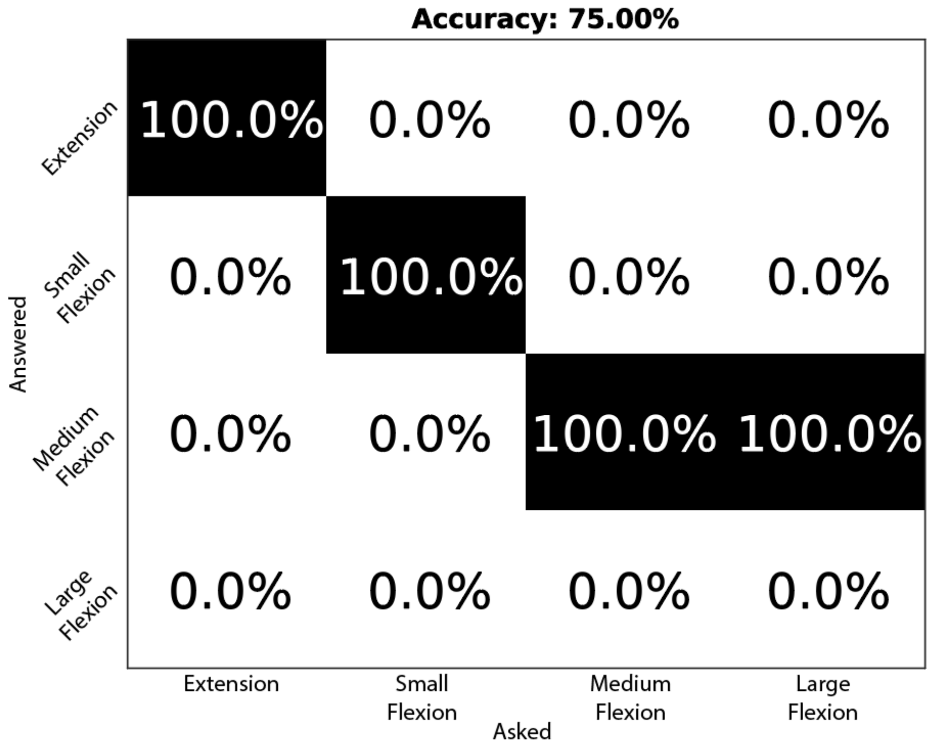

Figure S6. NI1 flexion level task performance with 4 levels. Corresponding levels of charge injected can be found in Table S2 in the CH 1 row.
